# Structural and functional identification of two distinct inspiratory neuronal populations at the level of the phrenic nucleus in the rat cervical spinal cord

**DOI:** 10.1007/s00429-018-1757-3

**Published:** 2018-09-24

**Authors:** Yoshio Shinozaki, Shigefumi Yokota, Fumikazu Miwakeichi, Mieczyslaw Pokorski, Ryoma Aoyama, Kentaro Fukuda, Hideaki Yoshida, Yoshiaki Toyama, Masaya Nakamura, Yasumasa Okada

**Affiliations:** 1 Department of Orthopaedic Surgery, School of Medicine, Keio University, 35 Shinanomachi, Shinjuku-ku, Tokyo, 160-8582 Japan; 2 Department of Anatomy and Neuroscience, Shimane University School of Medicine, 89-1 Enya-cho, Izumo, 693-8501 Japan; 3 Department of Statistical Modeling, The Institute of Statistical Mathematics, Tachikawa, Tokyo 190-8562 Japan; 4 Department of Statistical Science, School of Multidisciplinary Sciences, The Graduate University for Advanced Studies, Tachikawa, Tokyo 190-8562 Japan; 5 Laboratory of Electrophysiology Clinical Research Center and Division of Internal Medicine, Murayama Medical Center, Musashimurayama, Tokyo 208-0011 Japan; 6 Faculty of Physiotherapy, Opole Medical School, 68 Katowicka Street, Opole, 45-060 Poland; 7 Division of Pulmonary Medicine, Department of Internal Medicine, Keio University School of Medicine, Shinjuku-ku, Tokyo, 160-8582 Japan

**Keywords:** Astrocyte, Cervical spinal cord, Interneuron, Phrenic nucleus, Phrenic motoneuron, Respiratory control, Scalene motoneuron, Voltage imaging

## Abstract

The diaphragm is driven by phrenic motoneurons that are located in the cervical spinal cord. Although the anatomical location of the phrenic nucleus and the function of phrenic motoneurons at a single cellular level have been extensively analyzed, the spatiotemporal dynamics of phrenic motoneuron group activity have not been fully elucidated. In the present study, we analyzed the functional and structural characteristics of respiratory neuron population in the cervical spinal cord at the level of the phrenic nucleus by voltage imaging, together with histological analysis of neuronal and astrocytic distribution in the cervical spinal cord. We found spatially distinct two cellular populations that exhibited synchronized inspiratory activity on the transversely cut plane at C4–C5 levels and on the ventral surface of the mid cervical spinal cord in the isolated brainstem–spinal cord preparation of the neonatal rat. Inspiratory activity of one group emerged in the central portion of the ventral horn that corresponded to the central motor column, and the other appeared in the medial portion of the ventral horn that corresponded to the medial motor column. We identified by retrogradely labeling study that the anatomical distributions of phrenic and scalene motoneurons coincided with optically detected central and medial motor regions, respectively. Furthermore, we anatomically demonstrated closely located features of putative motoneurons, interneurons and astrocytes in these regions. Collectively, we report that phrenic and scalene motoneuron populations show synchronized inspiratory activities with distinct anatomical locations in the mid cervical spinal cord.

## Introduction

The diaphragm is the principal inspiratory pump muscle that is essential to maintain adequate ventilation in mammals, and its dysfunction elicits respiratory failure (Poole et al. 1997). It is innervated by the phrenic nerves, and the anatomical localization of the phrenic motoneuron pool in the cervical spinal cord has been investigated in various animal species (Keswani and Hollinshead 1955; Mitchell and Warwick 1956; Rao et al. 1972; Ullah 1978; Webber et al. 1979; Kuzuhara and Chou 1980; Rikard-Bell and Bystrzycka 1980; Goshgarian and Rafols 1981; Johnson and Getting 1988; Gordon and Richmond 1990) and in humans (Elliott 1942; Keswani and Hollinshead 1955; Hollinshead and Keswani 1956; Routal and Pal 1999). In addition, the somatic and dendritic morphology of phrenic motoneurons has been extensively investigated (Takahashi et al. 1980; Goshgarian and Rafols 1984; Rose et al. 1984; Takahashi and Ninomiya 1985; Furicchia and Goshgarian 1987; Anderson et al. 1988; Johnson and Getting 1988; Cameron et al. 1990, 1991; Lindsay et al. 1991; Monteau and Hilaire 1991; Torikai et al. 1996; Allan and Greer 1997; Prakash et al. 2000; Song et al. 2000; Okada et al. 2010; Lane 2011). Furthermore, electrophysiological properties of phrenic motoneurons have been investigated at a cellular level (Gill and Kuno 1963a, b; Berger 1979; St John and Bartlett 1985; Smith et al. 1988; Liu and Feldman 1992; Martin-Caraballo and Greer 1999, 2000, 2001; Cameron and Nunez-Abades 2000; Lee and Fuller 2011). Despite the abundance of the research on phrenic motoneurons, most studies are either purely anatomical or physiological at a single cellular level.

In the phrenic motoneuron pool, it is expected that not only motoneurons, but also interneurons play important roles in generation of inspiratory motor output (Bellingham and Lipski 1990), as in the locomotor central pattern generator of the lumbar spinal cord (Butt et al. 2002). Understanding the functional anatomy of the phrenic motoneuron pool is of significance not only in understanding the physiological function of phrenic motoneurons and their responses to chemical stimuli (e.g., to CO_2_/pH changes), but also for medical purposes. For example, precise knowledge of functional anatomy of the phrenic motoneurons is necessary for the exact diagnosis and planning of surgery of various diseases and injuries of the cervical spinal cord (Warren and Alilain 2014).

It has been elucidated that not only neurons, but astrocytes are actively involved in the formation of motor output in various regions of the central nervous system (Okada et al. 2012; Christensen et al. 2013). Furthermore, it has been recently clarified that intraspinal transplantation of human iPS cell-derived astrocytes preserve respiratory function after cervical spinal cord injury in rats and mice (Li et al. 2015). We also reported the importance of astrocytes in therapy of amyotrophic lateral sclerosis model mice by intraspinal transplantation of human iPS cell-derived glial-rich neural progenitors (Kondo et al. 2014). These reports suggest the existence of unexplored important significance of neuron–astrocyte interaction in the maintenance and recovery of motor function in the spinal cord.

Therefore, in the present study, we conducted detailed investigation of the spatiotemporal characteristics of inspiration-related neuronal activities and their responses to CO_2_/pH changes, generated in and around the phrenic motoneuron pool, by imaging with a fast-responding voltage-sensitive dye (voltage imaging) (Onimaru and Homma 2003; Yoshida et al. 2003; Fukuda et al. 2006; Okada et al. 2007; Oku et al. 2007, 2008; Aoyama et al. 2011; Koshiya et al. 2014; Iizuka et al. 2016). We also conducted detailed histological examination focusing on not only motoneurons, but interneurons and astrocytes in and around the phrenic nucleus. Furthermore, since the scalene muscle is an important accessory inspiratory muscle (Campbell 1955; Sant’ambrogio and Camporesi 1973; De Troyer and Estenne 1984; Fournier and Lewis 1996; Saboisky et al. 2007), innervated by branches of the cervical ventral rami (Sakamoto 2012), we presumed that scalene motoneurons, located near the phrenic nucleus in the cervical spinal cord, might show inspiratory-related activity. Thus, we also investigated the function and anatomy of the scalene motoneuron population, which, to the best of our knowledge, have not yet been documented.

## Materials and methods

All experiments were carried out in accordance with the National Institutes of Health Guide for the Care and Use of Laboratory Animals (NIH Publications No. 80-23) revised 1996 and with the Guiding Principles for the Care and Use of Animals of the Physiological Society of Japan. Experiments for voltage imaging and for anatomical analysis were approved by the Animal Experiment Ethics Committees of Keio University (Permit Number: 020062) and Shimane University (Permit Numbers: 03-34, H17-7, H19-53, H20-32 and IZ25-14). The study was conducted in neonatal Wistar rats of either sex.

### Brainstem–spinal cord preparation for voltage imaging

The brainstem and cervical spinal cord were isolated from the neonatal rat (*n* = 34 in total, 1–4 days) as previously described (Okada et al. 1993, 2005, 2007; Oku et al. 2007). Briefly, each animal was deeply anesthetized with diethyl ether, quickly decerebrated at the intercollicular level, and the brainstem and cervical spinal cord were together isolated. The cerebellum and brain structures rostral to the VIth cranial nerve root were removed, and the arachnoid membrane covering the medullary and spinal cord surface was carefully detached in a dissection chamber filled with mock cerebrospinal fluid (CSF; for contents, see below) that was equilibrated with 95% O_2_ and 5% CO_2_ at room temperature. For voltage imaging of the transversely cut surface, the cervical spinal cord was transected at the level between C4 and C5 segments with fine ophthalmologic scissors, and the preparation was incubated in oxygenated CSF containing a fast-responding voltage-sensitive dye di-4-ANEPPS (100–200 µg/ml; Invitrogen, Carlsbad, CA, USA) for 30 min (Yoshida et al. 2003; Fukuda et al. 2006; Aoyama et al. 2011; Koshiya et al. 2014). For imaging of the ventral surface, the spinal cord was transected at the C6 level and stained with a fast-responding voltage-sensitive dye di-2-ANEPEQ (50–100 µg/ml, Invitrogen) for 40 min (Okada et al. 2007; Oku et al. 2007, 2008). After staining with a dye, the preparation was rinsed in CSF for 15 min to eliminate any excessive dye and transferred to a recording chamber (volume 2 ml). For imaging of the transversely cut surface of the spinal cord, the brainstem was placed horizontally with the ventral side facing up and fixed with miniature pins on the chamber floor that was made of silicon resin. The spinal cord was bent upwardly, approximately at the C1–C2 level. A cubic block made of silicon resin was placed on the chamber floor to mechanically support the dorsal side of the spinal cord. Thus, transversely cut plane of the spinal cord was horizontally secured. For imaging of the ventral surface of the spinal cord, the entire preparation was horizontally placed with the ventral side up and fixed on the chamber floor. The recording chamber was continuously superfused with CSF at a rate of 3 ml/min. The temperature of the superfusate was controlled at 27 ± 1 °C. The control CSF contained (in mM): NaCl 124, KCl 5.0, CaCl_2_ 2.4, MgSO_4_ 1.3, KH_2_PO_4_ 1.2, NaHCO_3_ 26, glucose 30; it was equilibrated with 95% O_2_ and 5% CO_2_ (pH 7.4). When testing the effect of CO_2_/pH changes, preparations were superfused first with hypocapnic CSF equilibrated with 98% O_2_ and 2% CO_2_ (pH 7.8), which was subsequently replaced with hypercapnic superfusate equilibrated with 92% O_2_ and 8% CO_2_ (pH 7.2) (Okada et al. 1993, 2007; Kawai et al. 1996, 2006). Inspiratory-related neuronal output was monitored from the C4 ventral root (C4VR) with a glass suction electrode. The C4VR signal was amplified using a bioelectric amplifier (AB651J, Nihon Kohden, Tokyo, Japan), band-pass filtered (*λ* = 15 Hz–3 kHz), and integrated using a leaky integrator (EI601G, Nihon Kohden, Tokyo, Japan) with a time constant of 100 ms without rectification.

### Voltage imaging

Depolarizing activity either on the transversely cut surface or the ventral surface of the spinal cord (between the C3 and C5 levels) was recorded using an optical recording system MiCAM01 (BrainVision, Tokyo, Japan) (Yoshida et al. 2003; Fukuda et al. 2006; Aoyama et al. 2011). Briefly, preparations in the recording chamber that was placed under an epifluorescent microscope (Eclipse E600FN, Nikon, Tokyo, Japan) equipped with a 4× objective lens (Plan Apo, NA 0.2, Nikon) were illuminated through an excitation filter (*λ* = 535 ± 10 nm) with a tungsten–halogen lamp (250 W; Oriel, Stratford, CT, USA) driven by a stable DC power source (PD36-20; Kenwood, Tokyo, Japan). Epifluorescence through a barrier filter (long pass *λ* > 610 nm) was captured using a MiCAM01-CCD camera (spatial resolution 60 × 90 pixels). The change in fluorescence intensity (Δ*F*) relative to the initial intensity of fluorescence (*F*0) in each pixel was recorded at a rate of one frame/5 ms (total 681 frames) or one frame/20 ms (total 170 frames). In recording of a total of 3.41 s in both frame rates, C4VR activity was used to “pre-trigger” the recording system so that signals of C4VR activity and optical imaging data were recorded starting at 0.85 s before the onset of inspiratory C4VR activity.

The recording was repeated ten times at 10-s intervals, and the fluorescence signals were averaged across all repetitions. To normalize the difference in the amount of membrane-bound dye and illumination within the preparation, background fluorescence intensity at each pixel was divided by the maximal background fluorescence. Then the ratio of Δ*F* to the normalized background fluorescence intensity (*F*), i.e., the fractional change in fluorescence intensity (Δ*F*/*F*), was calculated at each pixel in each frame. If *F* was less than 0.25, then Δ*F*/*F* was set to be zero. A negative Δ*F*/*F* corresponds to membrane depolarization (Fukuda et al. 2006; Okada et al. 2007; Oku et al. 2007, 2008; Aoyama et al. 2011; Koshiya et al. 2014).

Voltage imaging was initiated, after we confirmed that inspiratory C4VR activity was stabilized while superfused with control or hypocapnic CSF (normally in 20 min after placing the preparation in a recording chamber). In the analysis of the effect of CO_2_/pH changes, voltage imaging was first conducted with hypocapnic CSF, and the superfusate was replaced with hypercapnic CSF. Voltage imaging with hypercapnic CSF was initiated in 5 min after superfusate replacement (Okada et al. 1993).

### Statistical analysis

To quantitatively examine the effects of hypercapnia on inspiratory depolarizing activities in the central and medial regions (details of these two regions are described later), the peak amplitudes and areas under the depolarizing wave curves between 0.84 and 2.5 s after the onset of recording in the hypocapnic condition were compared with those in the hypercapnic condition by a paired *t* test. The significance level was set at *p* < 0.05. For this purpose, signals in voltage imaging were pre-processed as follows: (1) visual identification of central and medial motor regions, (2) spatial averaging (3 pixels × 3 pixels binning), (3) removal of linear trend, (4) detection of the peak of inspiratory depolarization between 0.84 and 2.5 s after the onset of recording, and (5) integration of depolarizing inspiratory signals between 0.84 and 2.5 s after the onset of recording to obtain areas of depolarizing optical signals. The mathematical processing was performed using a software MATLAB (MathWorks, Natick, MA, USA). Data were presented as means ± SD.

### Retrograde tracing of phrenic and scalene motoneurons

We examined the anatomical distributions of phrenic and scalene motoneurons in the cervical spinal cord by retrograde tracing with fluorescent carbocyanine dye, 1,1′,dioctadecyl-3,3,3′,3-tetramethylindocarbocyanine perchlorate (DiI, Molecular Probes/Thermo Fisher Scientific, Eugene, OR, USA) (Ono et al. 1998). Application of DiI to the phrenic nerve (*n* = 17) or to the scalene muscle (*n* = 14) was made in diethyl ether anesthetized neonatal rats (1–2 days). To stain phrenic motoneurons, the proximal cut end of the right phrenic nerve was dipped in 10% DiI solution dissolved with dimethylformamide. To stain scalene motoneurons, three to five injections of DiI solution (0.05 µl/injection) were made into the right scalene muscle through a fine glass pipette attached to a 1.0 µl Hamilton microsyringe. After 24 h survival, the animals were deeply anesthetized with chloral hydrate (350 mg/kg), and fixed by transcardial perfusion with 5 ml of saline, followed by perfusion with 10 ml of 4% paraformaldehyde in 0.1 M phosphate buffer (PB, pH 7.3). The brainstems together with spinal cords were isolated, post-fixed overnight in 4% paraformaldehyde in PB and then saturated with a cold solution of 20% sucrose in PB. Subsequently, spinal cords were cut serially into frontal or horizontal sections of 50 µm thicknesses on a freezing or vibrating microtome. Sections were mounted onto gelatinized slides, and observed under an epifluorescent microscope (Eclipse E-800, Nikon) as well as under a confocal laser scanning microscope (FV300, Olympus, Tokyo, Japan).

### Histological examination of cell marker distributions

The distribution of neurons, putative motoneurons and astrocytes in the cervical spinal cord was examined. For this purpose we stained the cervical spinal cord tissue with cresyl violet (Nissl stain) that preferentially stains neurons (Gittins and Harrison 2004; Korzhevskii and Otellin 2005). In addition, we conducted immunohistochemistry for neuronal nuclear antigen (NeuN) for neurons, choline acetyltransferase (ChAT) for cholinergic cells to identify putative motoneurons, and glial fibrillary acidic protein (GFAP) as well as S100-protein β-subunit (S100) for astrocytes as previously described (Yokota et al. 2004, 2007, 2008, 2010; Aoyama et al. 2011; Koshiya et al. 2014). Briefly, neonatal rats (*n* = 5, 2–3 days) were deeply anesthetized with diethyl ether or chloral hydrate (700 mg/kg) and transcardially perfused with 10 ml of saline, followed by 20 ml of 4% paraformaldehyde or 10% formalin in 0.1 M PB. The spinal cords were isolated, post-fixed overnight in the same fixative at 4 °C, and then immersed in cold 20% sucrose in PB. Subsequently, the spinal cords (C3–C5 level) were cut into 40 or 50 µm thick transverse sections or 40 µm thick horizontal sections on a freezing microtome.

For light microscopic observation, sections were incubated in blocking solution composed of 3% normal donkey serum and 0.2% Triton-X in phosphate buffered saline (PBS, pH 7.3) for 30 min, and then incubated overnight in blocking solution containing mouse anti-NeuN (MAB377, EMD Millipore, Billerica, MA, USA; 1:100) or rabbit anti-S100 (ab41548, Abcam, Cambridge, UK; 1:500). For detection of GFAP, sections were incubated in sodium citrate buffer (10 mM, pH 6.0, 100 °C) for 15 min for antigen retrieval, and then incubated overnight in blocking solution containing mouse anti-GFAP (G3893, Sigma-Aldrich, 1:500). Subsequently, sections were incubated in blocking solution containing biotinylated donkey anti-mouse IgG (Jackson Immunoresearch Laboratories, West Groove, PA, USA; 1:500) for NeuN and GFAP or biotinylated donkey anti-rabbit IgG (Jackson Immunoresearch Laboratories; 1:500) for S100 for 4 h. Subsequently, the sections were incubated in PBS containing 0.2% Triton-X and avidin–biotin–peroxidase complex (Elite-ABC; Vector Laboratories, Burlingame, CA, USA; 1:1000) for 1 h, and then developed in 25 ml of 0.1 M PB containing 10 mg diaminobenzidine (DAB) and 10 µl of 30% hydrogen peroxide. For detection of ChAT, sections were treated with 1% H_2_O_2_ to inhibit intrinsic peroxidase activity, incubated overnight in blocking solution containing goat anti-ChAT (AB-144P, Chemicon; 1:1000), further incubated in blocking solution containing biotinylated donkey anti-goat IgG (Jackson Immunoresearch Laboratories; 1:500) for 4 h, and then in PBS containing 0.2% Triton-X and Elite-ABC for 1 h. Afterward, sections were reacted with biotin-conjugated tyramide (Perkin-Elmer Life Science, Waltham, MA, USA; 1:50) for 10 min. Subsequently, sections were incubated in PBS containing Elite-ABC for 1 h, and developed with DAB as outlined above. Finally, sections were mounted onto gelatinized slides, coverslipped with VectaMount (Vector Laboratories), and observed under a light microscope (Eclipse E800, Nikon). In the absence of primary antibody, no positive immunoreactivity was observed. Cytoarchitecture of the spinal cord was evaluated based on the anatomical atlas of the neonatal rat spinal cord (Paxinos et al. 1991).

For immunofluorescent observation, sections were treated with 1% H_2_O_2_, incubated in blocking solution containing antibody mixture of goat anti-ChAT (1:1000), rabbit anti-S100 (1:500), and either mouse anti-GFAP (1:500) or mouse anti-NeuN (1:100). Subsequently, sections were incubated in blocking solution containing biotinylated donkey anti-goat IgG, next in Elite-ABC (1:1000), and then reacted with Cy3-conjugated tyramide (Perkin-Elmer Life Science; 1:50) for 10 min. Sections were further incubated in blocking solution containing Alexa488-conjugated donkey anti-rabbit IgG (Molecular Probes/Thermo Fisher Scientific; 1:500) and Cy5-conjugated donkey anti-mouse IgG (Jackson Immunoresearch Laboratories; 1:500), mounted on gelatinized slides, counterstained with 4′,6-diamidino-2-phenylindole (DAPI; diluted with PBS at 1:2000; Dojindo, Kumamoto, Japan; catalog number FK045), and then coverslipped with VECTASHIELD (Vector Laboratories). Finally, the sections were observed under a confocal laser scanning microscope (FV1000, Olympus). The distributions of ChAT-immunoreactive (ir) neurons, S100-ir and GFAP-ir astrocytes, NeuN-ir neurons, and DAPI-positive nuclei were assessed from confocal images.

## Results

### Spatiotemporal activity of inspiratory-related depolarizing signals on the transverse cut plane

**Fig. 1:**
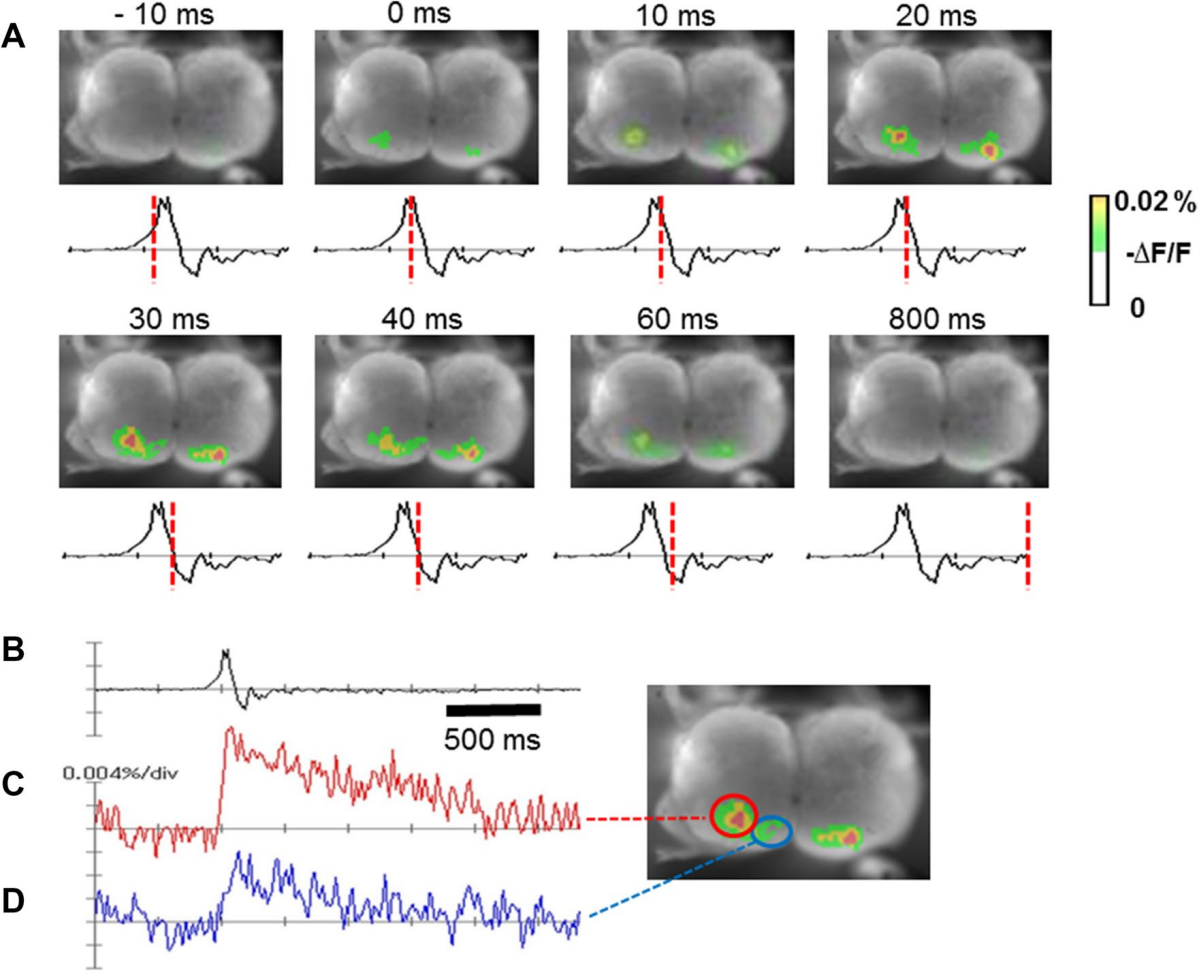
Inspiratory-related activity imaged on the transverse cut plane at the C4/C5 spinal cord of brainstem–spinal cord preparation superfused with normocapnic CSF. **a** Time course of optical inspiratory-related activity from − 10 to 800 ms. Traces below the optical images are integrated C4 inspiratory activity, where the peak of C4 inspiratory activity was defined as 0 ms, and red broken lines on these traces indicate the timing when the images shown above the traces were captured. **b** Integrated C4 activity. **c** Depolarizing optical signal in the central region. **d** Depolarizing optical signal in the medial region. Central and medial regions are indicated with red and blue circles, respectively, **b**–**d** were recorded simultaneously

In voltage imaging with 5 ms sampling rate on the C4/5 transverse plane of the cervical spinal cord with normocapnic CSF (*n* = 3), the depolarizing optical signal appeared in the ventral horn, rapidly expanding concentrically in the medial direction. The size of a depolarized region became maximum in approximately 10 ms after the peak of C4VR activity, which was followed by a gradual decrease during the inspiratory phase. Figure 1 shows representative optical images of the inspiratory-related depolarizing activity. We found that the inspiratory-related depolarizing region consisted of spatially distinct two subpopulations; one in the middle portion (central to the unilateral side) of the ventral horn corresponding to the central motor column and the other in the medial portion corresponding to the medial motor column. The activity appeared simultaneously in the central and medial portions of the ventral horn 20 ms before the peak of C4VR activity that was taken as 0 ms. Each activity gradually diminished with C4VR activity and disappeared in about 800 ms. Peak activity in the central portion appeared at about 20–30 ms after the peak of C4VR activity, and peak activity in the medial portion appeared at 40 ms after the peak of C4VR. The amplitude of the central portion activity was larger than that of the medial portion in each preparation. The finding that the inspiratory-related depolarizing region consisted of the spatially distinct two subpopulations was confirmed in other preparations in recording with 20 ms sampling rate (*n* = 4).

**Fig. 2:**
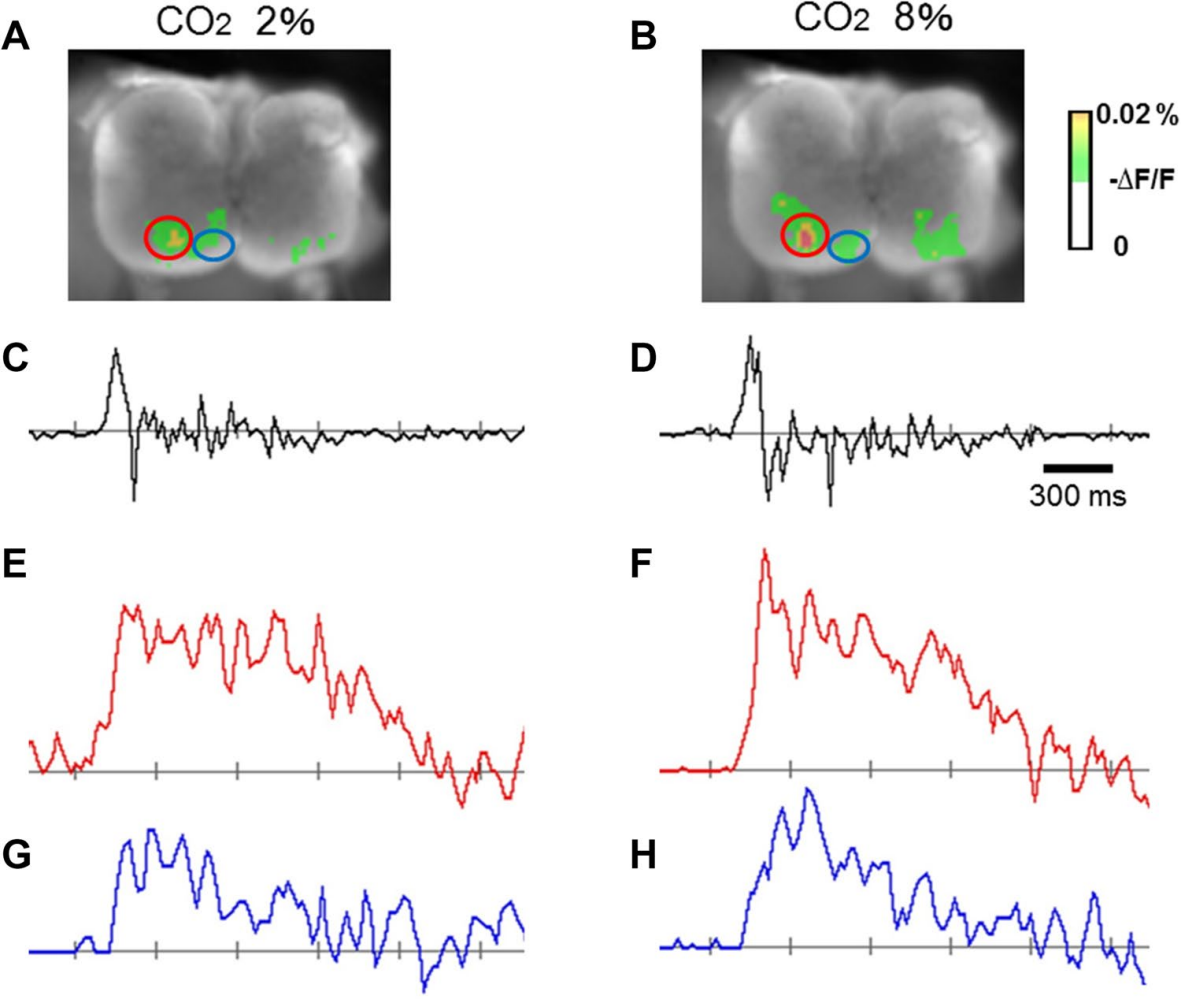
Representative depolarizing inspiratory optical signals obtained in the experimental hypocapnic (2% CO_2_) and hypercapnic (8% CO_2_) conditions. Changes in CO_2_ did not affect depolarizing inspiratory optical signals in either central or medial motor region. **a, b** Depolarizing optical signals on the transverse cut plane at C4/C5 level. Central and medial regions are indicated with red and blue circles, respectively. **c, d** Integrated C4 activity. **e, f** Depolarizing optical signals in the central region. **g, h** Depolarizing optical signals in the medial region, **a, c, e**, **g** correspond to hypocapnia, **b, d, f**, **h** correspond to hypercapnia

**Fig. 3:**
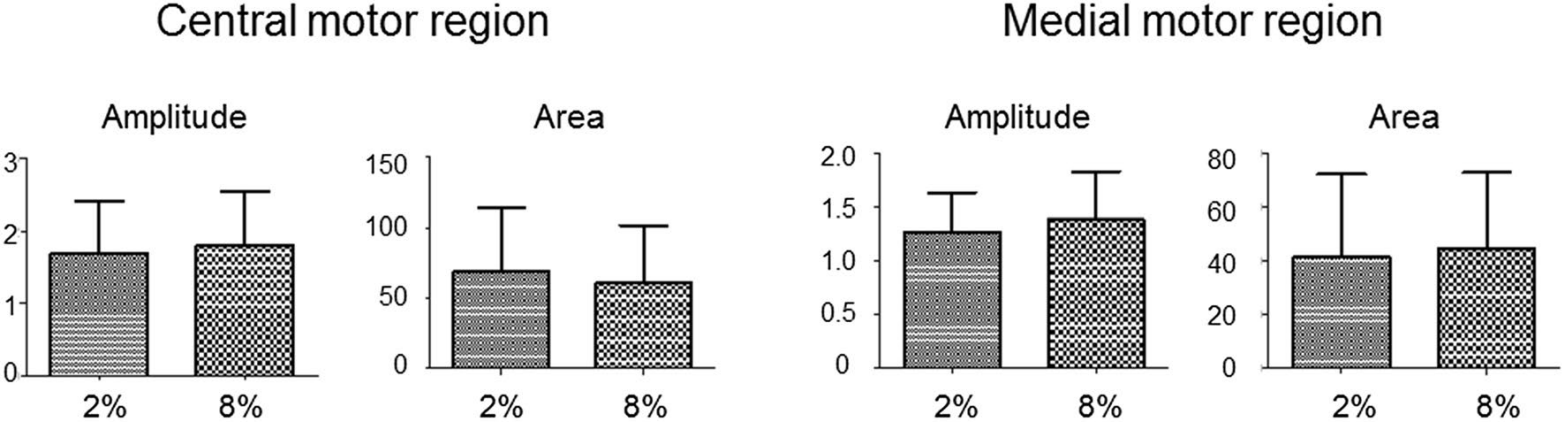
Comparison of depolarizing inspiratory optical signals obtained in hypocapnic (2% CO_2_) and hypercapnic (8% CO_2_) conditions (*n* = 13). Changes in CO_2_ did not appreciably affect the peak amplitude or area under the depolarizing wave curve between 0.84 and 2.5 s after the onset of the recording in either central or medial motor region. Each ordinate, arbitrary unit (au)

We examined the effects of hypercapnia on the inspiratory depolarizing activity in the central and medial regions on the cut surface at C4/5 level, corresponding to phrenic and scalene motor nuclei, respectively (the correspondence is explained later). Peak amplitude and area under the depolarizing wave curve were not appreciably different in either region and between the two regions in either hypocapnic or hypercapnic condition, as representative optical images of the respiratory-related neuronal activities (Fig. 2) and the group data show (*n* = 13) (Fig. 3).

### Spatiotemporal pattern of respiratory-related neuronal activities on the ventral surface of the cervical spinal cord

**Fig. 4:**
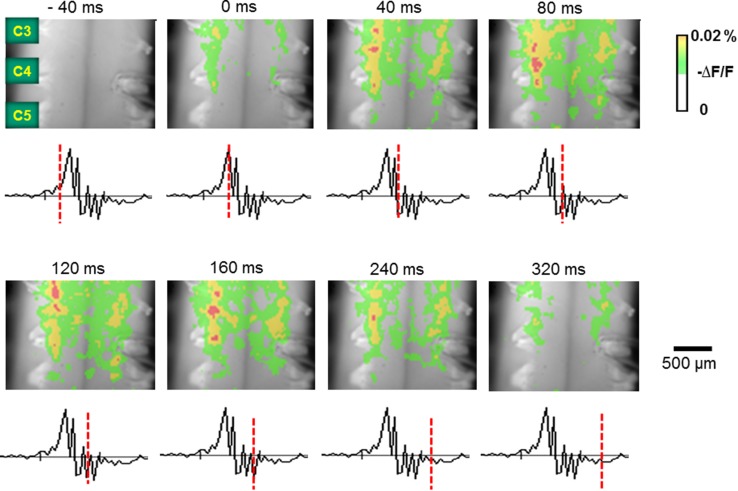
Inspiratory-related optical signals recorded from the ventral surface of the C3–C5 spinal cord superfused with normocapnic CSF, with C4 integrated activity and its time course from − 40 to 320 ms, where the peak of C4 inspiratory activity was defined as 0 ms. Two longitudinal columnar depolarizing regions, corresponding to central and medial regions, were observed

**Fig. 5:**
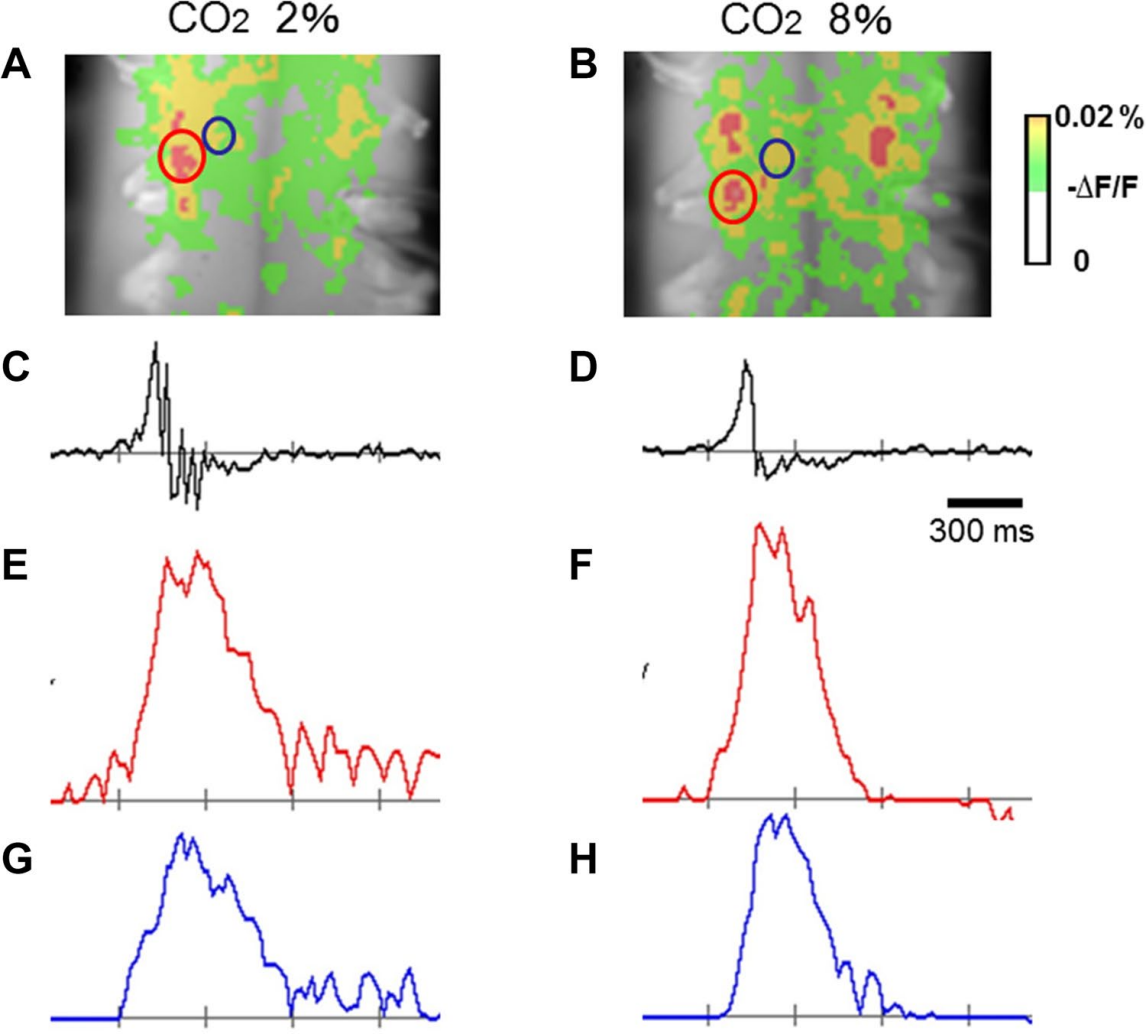
Representative optical images of inspiratory-related activity on the ventral surface of the cervical spinal cord in hypocapnic (2% CO_2_) and hypercapnic (8% CO_2_) conditions. In either central or medial motor region, changes in CO_2_ did not appreciably affect depolarizing inspiratory signals. **a, b** Images showing depolarizing optical signals on the ventral surface of the cervical spinal cord at C3–C5 level. Two longitudinal columnar depolarizing regions, corresponding to central and medial regions, were observed. Representative areas of central and medial regions for calculation of optical signal wave forms in **e**–**h** are indicated with red and blue circles, respectively. **c, d** Integrated C4 activity. **e, f** Depolarizing optical signals in the central region. **g, h** Depolarizing optical signals in the medial region. Each ordinate, arbitrary unit, **a, c, e**, **g** correspond to hypocapnia, **b, d, f**, **h** correspond to hypercapnia

Figure 4 shows representative optical images of the respiratory-related depolarizing activities on the ventral surface of the cervical spinal cord superfused with normocapnic CSF. Akin to the cut surface C4/5, we found that respiratory activities consisted of longitudinally distributed two subpopulations, i.e., inspiratory neuronal activities appeared as segmental clusters on the ventral surface in the central and medial portions, at C3–C5 levels. It must be noted that we did not conduct recording outside the region C3–C5. The activity first appeared in the medial portion of the ventral horn in C3 level 80 msec before the peak of C4VR activity, then extended rostro-caudally from C3 to C5. Another activity appeared in the central portion 60 ms before the peak of C4VR activity. The activity peaks in the medial and central portions at C3 level appeared 40–80 ms after the peak of C4VR activity, respectively. This spatiotemporal pattern of respiratory-related neuronal activities on the C3–C5 ventral surface was confirmed in all preparations (*n* = 8). Akin to the transverse cut plane, CSF change from hypocapnia to hypercapnia affected neither the amplitude nor the area of inspiratory depolarization in both central and medial motor regions (*n* = 6) (Fig. 5).

### Retrograde tracing of phrenic and scalene motoneurons

**Fig. 6:**
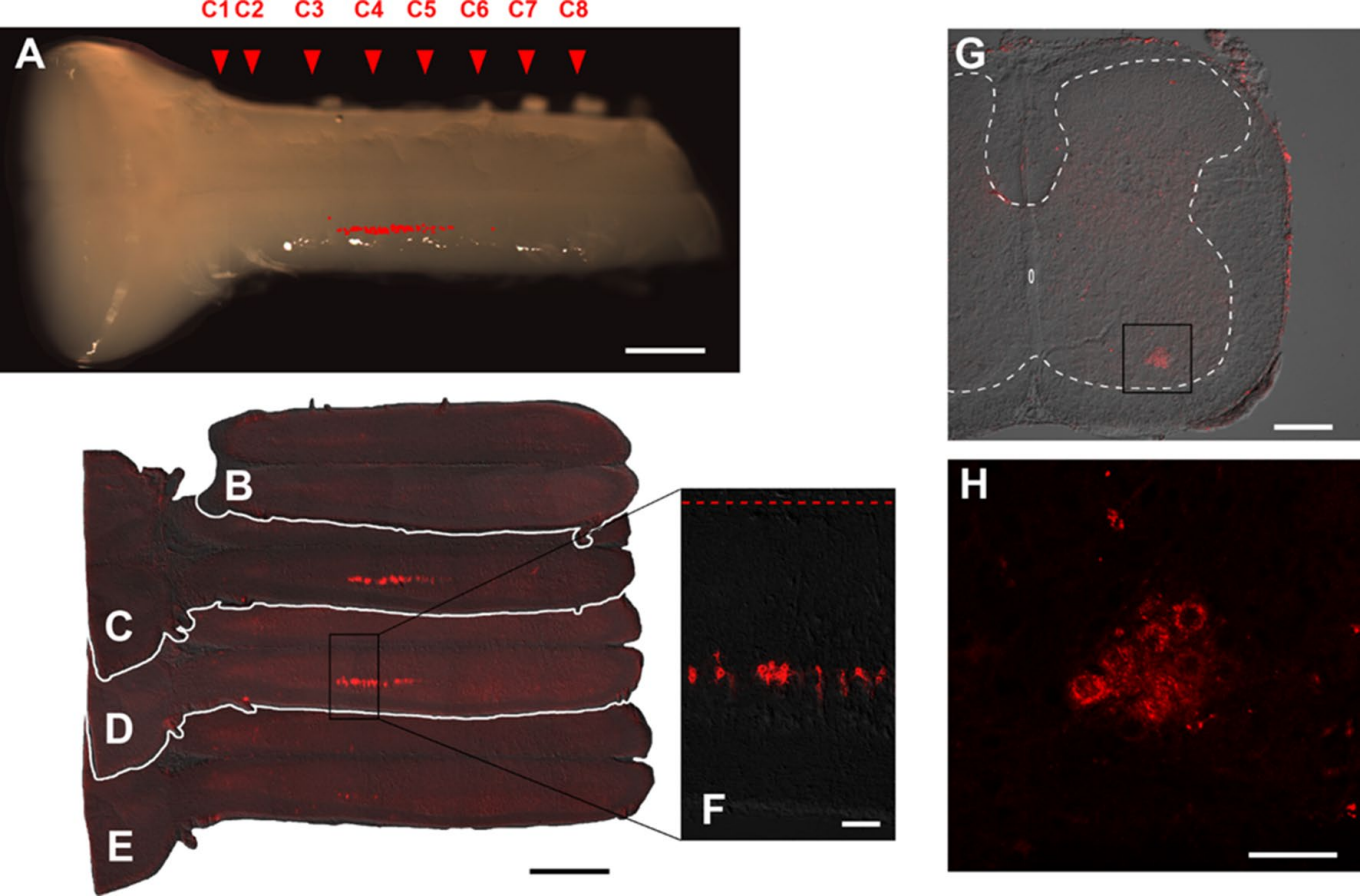
Photomicrographs and confocal images showing the distribution of DiI-labeled neurons in the spinal cord after DiI application to the phrenic nerve. **a** Ventral view of the brainstem–spinal cord. Location of DiI-labeled neurons is marked in red in the photomicrograph. **b**–**e** DiI-labeled neurons in horizontal section of the spinal cord (**b–e** ventral to dorsal). Red broken line in **f** indicates the midline of the spinal cord. **g, h** DiI-labeled neurons in transverse section of the spinal cord. The area enclosed with a rectangle in **g** is shown at higher magnification in **h**. Phrenic motoneurons are located in the central motor region between the C3 and C5 levels. Scale bars, **a**–**e** 1 mm; **f** 100 µm; **g** 200 µm; **h** 50 µm

**Fig. 7:**
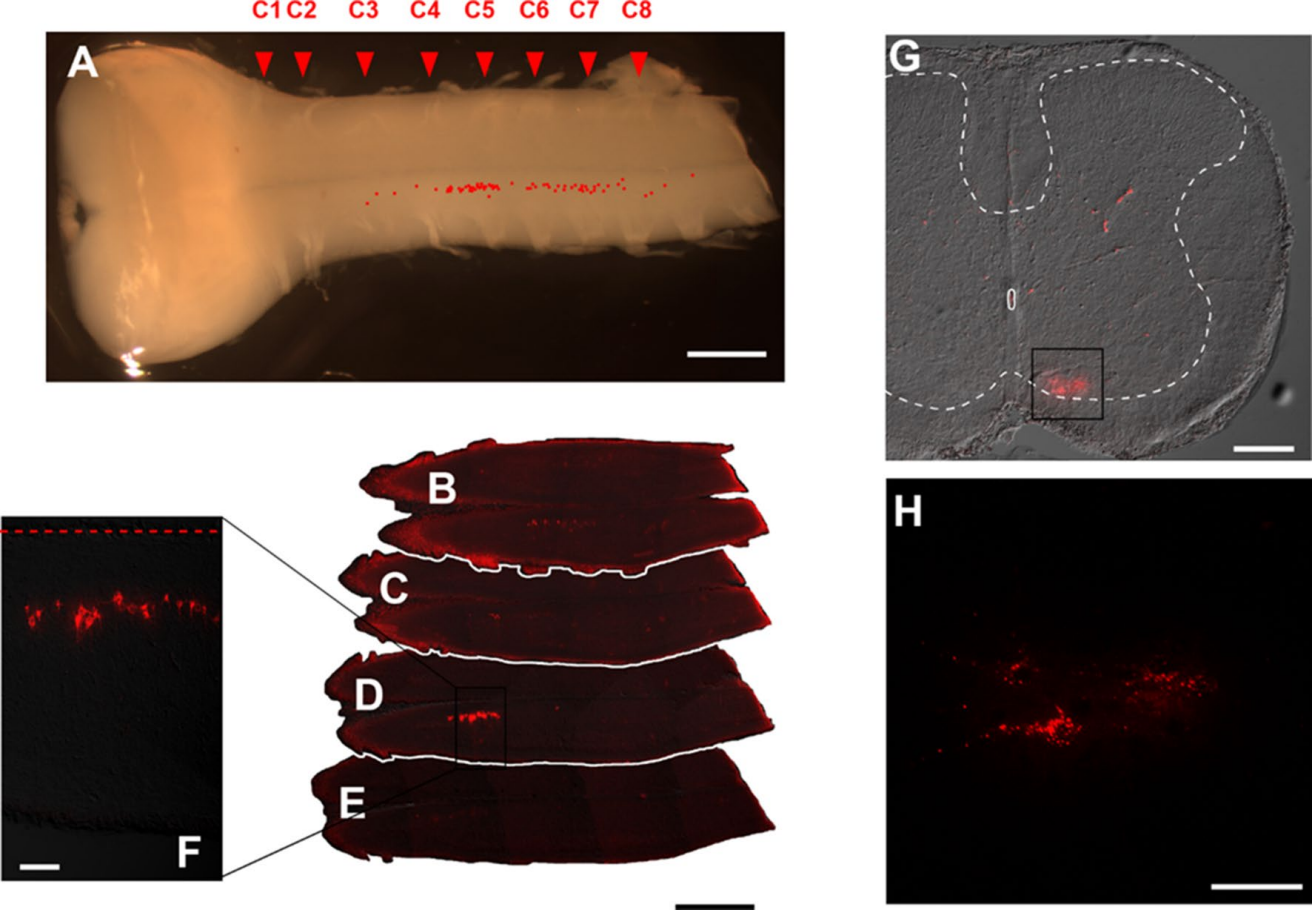
Photomicrographs and confocal images showing the distribution of DiI-labeled neurons in the spinal cord after DiI injection into the scalene muscle. **a** Ventral view of the brainstem–spinal cord. Location of DiI-labeled neurons is marked in red in the photomicrograph. **b–e** DiI-labeled neurons in horizontal section of the spinal cord (**b–e** ventral to dorsal). Red broken line in **f** indicates the midline of the spinal cord. **g, h** DiI-labeled neurons in transverse section of the spinal cord. The area enclosed with rectangles in **g** is shown at higher magnification in **h**. Distribution of scalene motoneurons is longer (between the C3 and C5 levels) and more medial as compared to that of phrenic motoneurons as shown in Fig. 8. Scale bars, **a**–**e** 1 mm; **f** 100 µm; **g** 200 µm; **h** 50 µm

After DiI application to the phrenic nerve, DiI-labeled neurons were observed in the central motor column region of the ventral horn in C3–C5 segments (Fig. 6). After DiI injection into the scalene muscle, DiI-labeled neurons were observed in the medial motor column region of the ventral horn in C3–C8 segments (Fig. 7). Anatomical distributions of DiI-labeled phrenic and scalene motoneurons coincided with those of optically detected regions in the central and medial portions of the ventral horn, respectively.

**Fig. 8:**
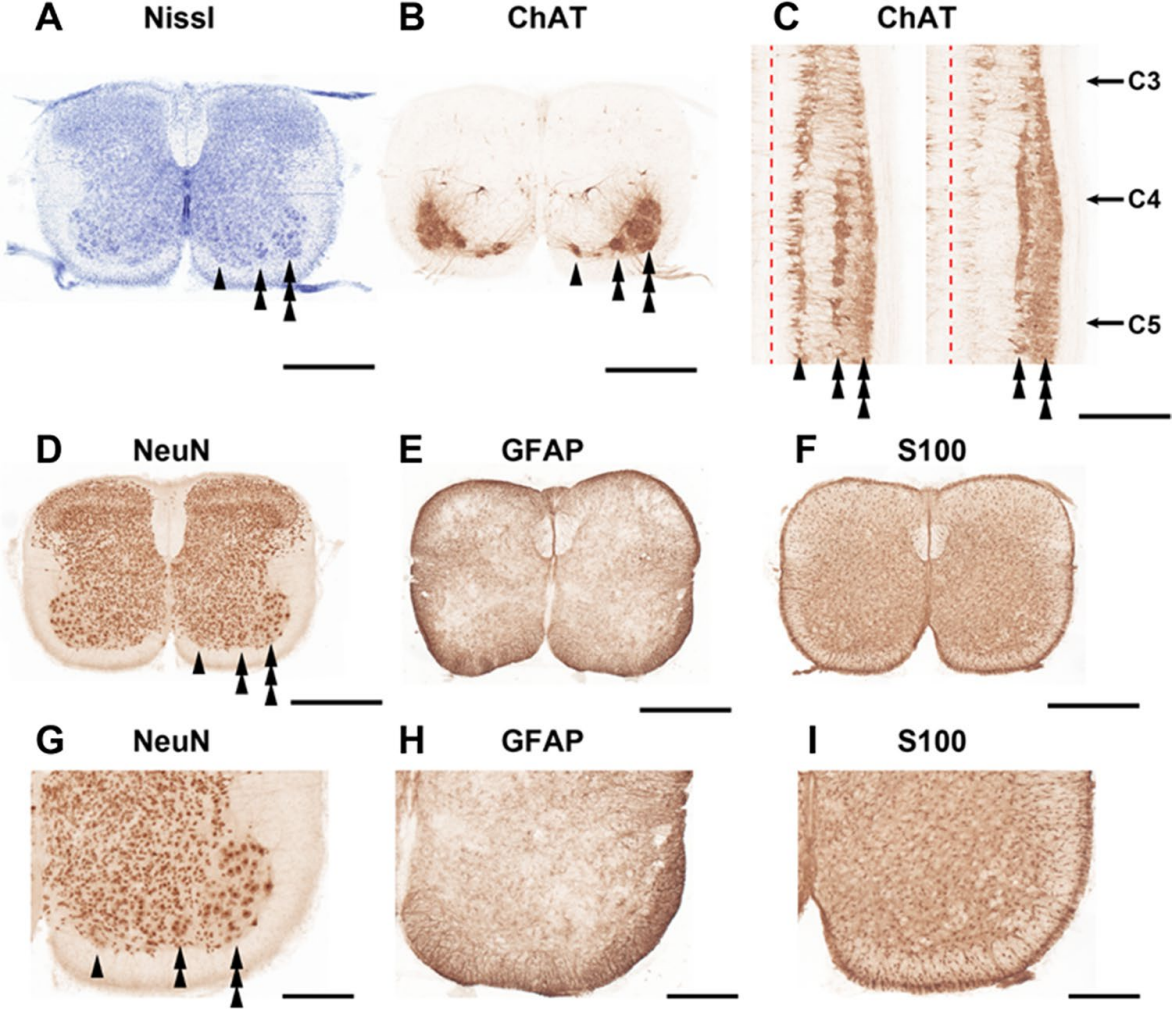
Photomicrographs taken at C4 level of the cervical spinal cord. **a** Nissl-staining. **b** Choline acetyltransferase (ChAT)-immunoreactive neurons. **c** ChAT-immunoreactive neurons in the horizontal section of the ventral horn of C3–C5 spinal cord. Red broken line indicates the midline of the spinal cord. **d–f** Immunostaining of neuronal nuclear antigen (NeuN), glial fibrillary acidic protein (GFAP) and S-100-protein β-subunit (S100), respectively. **g–i** Enlarged images of the **d**–**f**, respectively. Arrowheads, double-arrowheads, and triple-arrowheads indicate the medial, central, and lateral motor columns, respectively. Scale bars: **a**–**f** 500 µm; **g**–**i** 200 µm

### Histological examination of cell marker distributions

Figure 8 shows the photomicrographs of the transverse plane of the spinal cord at the C4 level with Nissl-staining, and immunostainings of ChAT, NeuN, GFAP, and S100. Nissl-staining showed diffuse distribution of cell bodies in the gray matter, with particularly high density in the ventral horn (laminae IX) (Fig. 8a). ChAT-ir neurons in the C4 cervical spinal cord were located in the most ventral layer of the ventral horn (Fig. 8b). A subpopulation of ChAT-ir neurons in the ventromedial portion of the ventral horn, which constitutes the medial motor column, were fusiform in shape and located along the ventral funiculus. In the ventrolateral portion of the ventral horn, a number of large-sized, polygonal-shaped ChAT-ir neurons were located, corresponding to the lateral motor column. A population of multipolar ChAT-ir neurons was confined to the mid-ventral portion of the ventral horn, which corresponds to the central motor column. On the horizontal plane of the ventral horn, three longitudinal clusters of ChAT-ir neurons were found. The neurons in the ventromedial and ventrolateral portions of the ventral horn were distributed through the entire rostro-caudal levels of the cervical spinal cord, while those in the mid-ventral portion were seen only between C3 and C5 levels (Fig. 8c).

Distribution of immunoreactivity for NeuN was akin to that of Nissl-positive cells, but the laminar structure of the gray matter, especially in the dorsal horn, was more clearly demonstrated by NeuN immunocytochemistry than by Nissl-staining (Fig. 8d, g). GFAP-ir was strong in the white matter, especially in the marginal layer, and GFAP-ir cells with fibrillary processes, apparently fibrous astrocytes, were distributed in the whole gray matter (Fig. 8e, h). Regarding another astrocytic marker, S100-ir cells were distributed roughly equally in the gray and white matter, except for the marginal layer, where S100-ir was particularly strong (Fig. 8f, i).

**Fig. 9:**
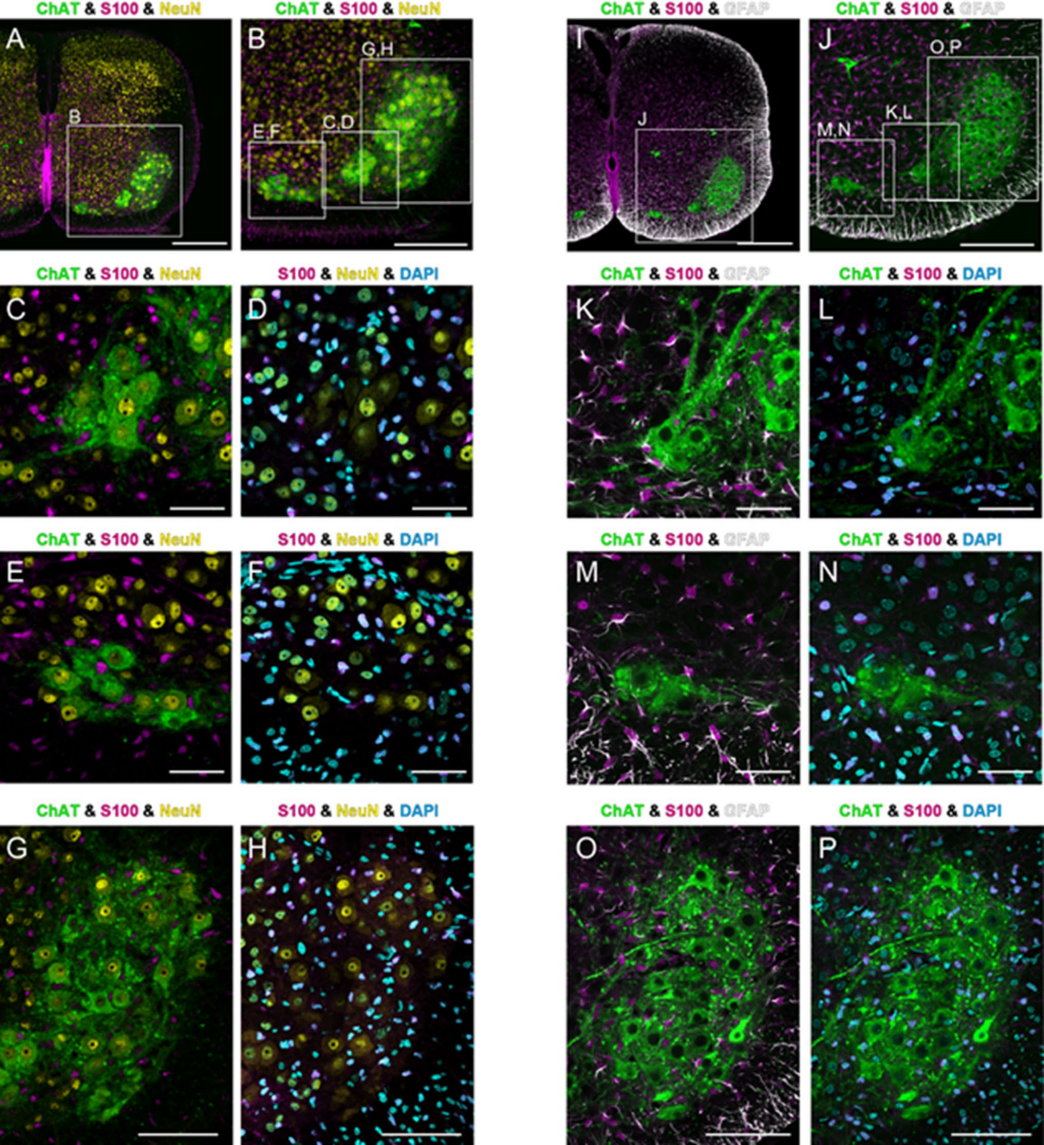
Confocal images showing distribution of immunoreactivity of various cell markers at C4 level of the cervical spinal cord (**a, i**). The areas enclosed with rectangles in **a** and **i** are shown at higher magnifications in **b** and **j**, respectively. The central, medial, and lateral motor columns enclosed with rectangles in **b, j** are shown at higher magnification in **c, d, k, l** and **e, f, m, n** and **g, h, o, p**, respectively. Immunoreactivities for ChAT, S100, GFAP, and NeuN are indicated in green, magenta, white and yellow, respectively, and DAPI-positive cell nuclei are indicated in cyan. **c, e, g, k, m**, and **o** are same area as **d, f, h, l, n**, and **p**, respectively. Putative phrenic and scalene motoneurons (ChAT-ir large neurons in the central and medial motor regions, respectively) as well as motoneurons in the lateral motor region are surrounded by interneurons (ChAT-negative and NeuN-positive cells) and astrocytes (GFAP-ir or S100-ir cells). Scale bars: **a, i** 300 µm; **b, j** 200 µm; **c**–**f, k**–**n** 50 µm; **g, h, o, p** 100 µm

In the quadruple staining of various cell markers (ChAT, NeuN, GFAP, S100 and DAPI) in the ventral horn, we were able to distinguish ChAT-ir motoneurons, ChAT-negative NeuN-ir-positive neurons, astrocytes with GFAP- and S100-ir, and other cells stained solely with the nuclear DAPI marker (Fig. 9). In the medial column of the ventromedial portion, the central column of the mid-ventral portion, and the lateral column of the ventrolateral portion of the ventral horn, we found that GFAP-ir and/or S100-ir cells were intermingled with ChAT-ir neurons, and GFAP-ir fibrillary processes extending from S100-ir cells were associated with ChAT-ir motoneurons. Furthermore, ChAT-ir neurons constituting the medial and central motor columns were surrounded by a large number of ChAT-negative NeuN-ir positive cells.

## Discussion

The present study demonstrates that the respiratory neuronal population at the level of the phrenic nucleus in the cervical spinal cord consists of functionally and anatomically distinct two subgroups. The inspiratory activity appeared in the central portion of the ventral horn, almost simultaneously the other activity emerged in the medial portion, and both activities extended rostro-caudally within the imaged C3 and C5 regions. The two portions, central and medial, coincided with the anatomically identified phrenic and scalene motoneuron groups, respectively. The central and medial subpopulations, should be differently involved in respiratory output formation. Phrenic and scalene motoneurons have closely related, but distinct spatial activities. Changes of superfusate CO_2_ content did not affect the spatiotemporal inspiratory activity patterns either in the phrenic or scalene motor region.

### Localization and activity patterns of the phrenic and scalene motor neuron groups

It is well known that motoneurons in the spinal cord form longitudinal columnar clusters in the ventral horn, and they are typically classified as the medial, central, and lateral groups (Barber et al. 1984; Molander et al. 1989). Elliott (1942), and Keswani and Hollinshead (1955) reported that phrenic motoneurons are located in the most medial portion of the ventral horn in humans. Thereafter, a large number of anatomical studies reported the localization of the phrenic nucleus on the transverse plane of the cervical spinal cord in various animals (Ullah 1978; Warwick and Mitchell 1956; Webber et al. 1979).

The longitudinal distribution of the phrenic nucleus was reported as the region between C3 and rostral edge of C6 segments in the macaque by Warwick and Mitchell (1956) and between C4 and C6 segments in the rabbit by Ullah (1978). Regarding the DiI-labeling technique, it must be noted that DiI does not label neurons trans-synaptically (Bader et al. 2012). Thus, labeling of sensory afferent fibers should be limited within the primary neuron, and secondary relay neurons in the spinal cord should not be labeled. Indeed, we never found DiI-labeled neuronal somata in the dorsal horn. Furthermore, it must be noted that sensory afferents of the phrenic nerve in the spinal cord are distributed mainly not in the ventral, but in the dorsal horn (Goshgarian and Roubal 1986). Actually, we did not detect DiI-labeled fibers in the dorsal horn, presumably due to weakness of thin fiber labeling with DiI. Therefore, our DiI-labeling analysis clearly indicates that the phrenic motoneurons are located in the central motor region at the C3–C5 segments in the rat (Fig. 6). The descending inspiratory optical signals in our voltage imaging are compatible with anatomically observed dense rostro-caudal longitudinal projections of dendrites from phrenic motoneurons (Cameron et al. 1983).

The scalene muscle is an important accessory inspiratory muscle (Campbell 1955; Sant’ambrogio and Camporesi 1973; De Troyer and Estenne 1984; Fournier and Lewis 1996; Saboisky et al. 2007). However, to the best of our knowledge, the anatomy and function of the scalene motoneurons have never been studied, although the anatomy of motoneurons innervating other neck muscles has been well documented (Brichta et al. 1987). Therefore, the present study is the first documentation of the scalene motoneuron anatomy. Although there is no report to directly compare the muscle spindle densities in the diaphragm and scalene muscle, the scalene muscle may contain more muscle spindles than the diaphragm per unit volume (Critchlow and von Euler 1963; Fournier and Lewis 1996). As a result, when DiI is injected into the scalene muscle, both alpha and gamma motoneurons should be labeled in the scalene motor pool, and at least some DiI-labeled neurons observed in our study should be gamma motoneurons innervating the scalene muscle (Fig. 7). It is reported that the scalene muscle is active during the inspiratory phase in quiet breathing as well as in hypercapnia and in a mechanical stress-loaded condition. The functional role of scalene gamma motoneurons may be a positive feedback control of inspiratory neural output formation (Critchlow and von Euler 1963), which needs further investigation. The inspiratory activity pattern of the scalene muscle is similar to that of the diaphragm as shown with electromyography in the dog (D’Angelo et al. 1988; D’Angelo and Bellemare 1990). This similarity of inspiratory activity of the diaphragm and scalene muscle is in accordance with similar temporal activity patterns of central and medial regions in the present voltage imaging study. We did not observe a difference in inspiratory activities in voltage imaging between hypocapnic and hypercapnic conditions either in the phrenic or scalene motor region.

### Crossing phenomenon

The depolarizing optical signals medial to the phrenic motoneuron pool may be partly arising from the decussation fiber activity from the contralateral side (Goshgarian et al. 1991) and from the ventromedial dendritic bundle of phrenic motoneurons crossing the midline often seen in neonatal or juvenile, but not in adult rats (Furicchia and Goshgarian 1987; Lindsay et al. 1991; Allan and Greer 1997; Prakash et al. 2000; Song et al. 2000; Huang and Goshgarian 2009). These midline crossing dendrites reach the contralateral ventral horn, but never extend to the contralateral phrenic motoneuron pool, suggesting that they are not directly innervated by contralateral mutually excited phrenic motoneurons (Lindsay et al. 1991; Prakash et al. 2000). Depolarizing optical signals observed in the medial motor region in the present study are considered generated by scalene motoneurons, but also may partly reflect the activity of the crossed phrenic phenomenon that is particularly active in neonates (Zimmer and Goshgarian 2005; Huang and Goshgarian 2009). At the same rostro-caudal level with phrenic motoneurons, brachial motoneurons are also located. However, brachial motoneurons are positioned laterally to phrenic motoneurons (see Fig. 6 of Allan and Greer 1997). Therefore, the central and medial regions do not correspond to brachial motoneurons.

### Interneuron

Bellingham and Lipski (1990) mapped the locations of respiratory neurons at C5 level of the cat spinal cord, and found a number of inspiratory interneurons around the phrenic nucleus. Lane et al. (2008) and Qiu et al. (2010) reported the existence of phrenic interneurons. However, their localization was, apart from the phrenic nucleus, mainly in the ipsilateral region around the central canal. As Bellingham and Lipski (1990) reported, it is expected that a large number of inspiratory interneurons exist within the phrenic nucleus. However, due to technical limitations, the presence of interneurons within the phrenic nucleus has not been thoroughly studied either physiologically or anatomically (Lane 2011). Recently, Streeter et al. (2017) combined the techniques of multielectrode array recording and histochemistry, and mapped interneuron populations in the mid cervical spinal cord in adult rats. They suggested that a number of interneurons generate synchronous respiratory discharge with mono- and polysynaptic connections among neurons. In the present study we have histologically demonstrated that putative phrenic and scalene motoneurons (ChAT-ir large neurons in the central and medial motor regions, respectively) are surrounded by putative interneurons (ChAT-negative, NeuN-positive cells) (Fig. 9). Inspiratory depolarizing signals observed by voltage imaging in both central and medial motor areas in the present study could be attributed to inspiratory activity of not only motoneurons, but interneurons. Our finding is compatible with the notion by Streeter et al. (2017), and it is considered that the motoneurons and interneurons together form inspiratory neural output patterns in the phrenic and scalene microcircuits.

### Involvement of glial cells

In the present study, GFAP-ir and S100-ir cells were relatively sparse in the gray matter, compared to their dense distribution in the white matter of the cervical spinal cord (Figs. 8, 9). However, in the ventral horn, GFAP-ir and S100-ir cells were densely distributed in the two regions that correspond to the inspiratory regions detected by voltage imaging (Figs. 1, 8h, 9), although we also observed intermingled GFAP-ir and S100-ir cells in the lateral motor region that is non-respiratory (Fig. 9). It has been reported that astrocytes are actively involved in various aspects of respiratory control such as rhythm generation and prevention of hypoxic ventilatory depression (Okada et al. 2012; Fukushi et al. 2016; Sheikhbahaei et al. 2018). It has been reported that phrenic motoneurons show a close anatomical coupling with astrocytic processes (Goshgarian and Rafols 1984). These previous reports, our present findings, and our previous observation that activation of not only neurons, but astrocytes induce depolarizing optical signals in voltage imaging (Aoyama et al. 2011) collectively suggest the active involvement of astrocytes in inspiratory pattern formation in the phrenic nucleus. Notably, the long-lasting component of inspiration-synchronized depolarizing optical signals, which is longer than the activity of C4VR (Fig. 1b–d), might be attributed to neuroplastic action of astrocytes that are generally long-acting when once activated (Henneberger et al. 2010). Furthermore, it has been demonstrated that morphology of astrocytes in the phrenic nucleus changes when the spinal cord tissue rostral to the phrenic nucleus is injured or exposed to chronic hypoxia, suggesting the involvement of astrocytes in the neural plasticity of the phrenic motoneuron function (Goshgarian et al. 1989; Goshgarian and Yu 1990; Windelborn and Mitchell 2012). The present anatomical observations support the idea that astrocytes in the phrenic nucleus are involved in inspiratory pattern formation and its neural plasticity.

### Future perspective

The present study clarified the distinct spatiotemporal dynamics and their cellular composition of the phrenic and scalene motor pools in the cervical spinal cord. Further studies are necessary to elucidate the detailed functional interaction among motoneurons, interneurons and astrocytes in the phrenic and scalene motor neuron pools to understand the precise mechanism of respiratory output formation in the cervical spinal cord. These issues ought to be explored with other study designs, e.g., analysis of individual cell activities in the respiratory motor pools by calcium imaging, selective stimulation/inhibition of certain types of cells (neurons/astrocytes) by optogenetics, and genetic manipulation and pharmacological intervention (Okada et al. 2012; Tanaka et al. 2012; Sheikhbahaei et al. 2018). Furthermore, the present study would provide essential basic knowledge of anatomy and function for the neural stem cell transplantation in the future regenerative medicine in patients with cervical spinal cord injuries and diseases such as amyotrophic lateral sclerosis (Nakamura and Okano 2013; Kondo et al. 2014; Matsui et al. 2014).
